# Effective Use of Empirical Data for Virtual Screening against APJR GPCR Receptor

**DOI:** 10.3390/molecules26164894

**Published:** 2021-08-12

**Authors:** Laura C. E. Manoliu, Eliza C. Martin, Adina L. Milac, Laurentiu Spiridon

**Affiliations:** Department of Bioinformatics and Structural Biochemistry, Institute of Biochemistry of the Romanian Academy, Splaiul Independenţei 296, 060031 Bucharest, Romania; laura.manoliu@biochim.ro (L.C.E.M.); eliza.martin@biochim.ro (E.C.M.); amilac@biochim.ro (A.L.M.)

**Keywords:** structural bioinformatics, molecular docking, Alzheimer, apelin receptor

## Abstract

Alzheimer’s disease is a neurodegenerative disorder incompatible with normal daily activity, affecting one in nine people. One of its potential targets is the apelin receptor (APJR), a G-protein coupled receptor, which presents considerably high expression levels in the central nervous system. In silico studies of APJR drug-like molecule binding are in small numbers while high throughput screenings (HTS) are already sufficiently many to devise efficient drug design strategies. This presents itself as an opportunity to optimize different steps in future large scale virtual screening endeavours. Here, we ran a first stage docking simulation against a library of 95 known binders and 3829 generated decoys in an effort to improve the rescoring stage. We then analyzed receptor binding site structure and ligands binding poses to describe their interactions. As a result, we devised a simple and straightforward virtual screening Stage II filtering score based on search space extension followed by a geometric estimation of the ligand—binding site fitness. Having this score, we used an ensemble of receptors generated by Hamiltonian Monte Carlo simulation and reported the results. The improvements shown herein prove that our ensemble docking protocol is suited for APJR and can be easily extrapolated to other GPCRs.

## 1. Introduction

Alzheimer’s Disease (AD) is the most frequent neurodegenerative cause of dementia and is responsible for nearly three-quarters of dementia cases [1,2]. Most Alzheimer’s related research emphasizes the significance of amyloid beta (Aβ) accumulation in disease onset and development [3]. However, other elements can be considered: gene mutations, oxidative stress, inflammation, neurofibrillary tangles (NFT) accumulation, hormone imbalances, mitochondrial dysfunction, together with aging, hypertension, dyslipidemia and diabetes [4,5]. Here we focus on apelin receptor (APJR), a class A (rhodopsin-like) G-protein coupled receptor (GPCR) as apelin could be involved in preventing the production of Aβ and the accumulation of other proteins involved in the development of Alzheimer’s disease [6] and the observed high presence in the central nervous system (CNS).

APJR is encoded by the APLNR gene and activated by its endogenous peptidic ligand, apelin. Due to its affinity to the various forms of apelin (apelin-13, apelin-17 and apelin-36) and also because of its co-interaction with different G proteins, the APJ receptor contributes to the activation of many signaling pathways, causing various effects at physiological level, such as vasoconstriction and vasodilation, angiogenesis, regulation of energy metabolism and fluid homeostasis [7,8,9]. The APJR is also involved in pathologies such as cardiovascular disease, diabetes, obesity and cancer, making it a promising research target [10]. As to its importance in neurologic conditions, several studies were conducted in order to evaluate the distribution of the APJR receptor in humans [11,12,13]. mRNA expression has shown strong signals in the caudate nucleus, corpus callosum, hippocampus, gray matter, subthalamic nucleus and spinal cord [12]. Recently, the expression of mRNA has been revealed in the cortex, as well as in the hippocampus [12], although the function of the APJ receptor in most regions of the brain is still unclear. Its ligand, apelin, is known for its implications in neuroprotection, as well as in memory and learning and the prevention of neuronal damage [11]. Understanding APJR at a molecular level can lead to valuable applications and to this end in silico techniques such as molecular simulations are essential.

Molecular simulation has become an indispensable tool for understanding the conformational space, dynamics and interactions of biomolecules. In 2017, the complete APJR receptor in complex with its ligand was crystalized giving detailed insight into their molecular structure and interaction [14]. A couple of molecular dynamics simulations were conducted on APJR-apelin complex which led to better understanding of their interactions and dynamics [14,15]. We also use in silico approaches here, to sample APJR binding site conformations from their Boltzmann distribution using both Markov Chain Monte Carlo (MCMC) and molecular dynamics methods in a comparative fashion.

APJR is a transmembrane protein with 7 helical segments and its binding site exposed to the extracellular space. Simulating transmembrane proteins require the presence of a surrounding membrane otherwise they become unstable due to the exposed hydrophobic amino acids. The protein-membrane system, in turn, involves explicit solvation leading to large systems which require considerable computer effort in simulations. For this reason, a frequent choice to approximate the Boltzmann distribution is the usage of molecular dynamics. In our work, however, we also used a Hamiltonian Monte Carlo (HMC) simulation, which draws from high probability regions after a short burn-in but may be slower to converge. Our goal was not to reach convergence in the whole conformational space of the molecule, but rather to sample the amino acids of the binding sites around their minimum.

Hamiltonian/Hybrid Monte Carlo (HMC), in brief, is an MCMC method which uses a molecular dynamics trajectory to generate a proposal move [16,17]. The configurations are recovered by marginalization over the auxiliary momentum variable because they are not conditioned by the latter. The approach is highly popular due to the ability of performing large moves in the state space. In theory the Hamiltonian propagation preserves the energy and the efficiency is only dependent on the MCMC parameters, however, in practice, the integration is performed numerically and introduces errors leading to a decrease in its efficiency.

Molecular dynamics uses Newton’s laws of motion to generate trajectories in the phase space. In contrast with most of MCMC methods, it doesn’t have to obey detailed balance, therefore avoids a random-walk behaviour but it is also limited by the numerical integration which forces a maximum timestep.

The pool of resulting conformations was then used to improve a virtual drug screening procedure. A typical virtual screening protocol in the target-to-hit phase of a drug design pipeline consists in the preparation of the receptor to which is screening against, the preparation of a library of compounds drawn from a pool of candidates, such as ZINC database [18,19,20], an initial molecular docking step (Stage I) to find the best pose, a rescoring stage (Stage II) that roughly ranks the compounds to decrease the size of the library and a final free energy estimation step (Stage III) which yields the final ranking of the candidates [21,22]. Stage II and III are the most computationally expensive and our work attempts to reduce the effort by filtering and reducing the set that enters these stages.

Ensemble docking comes to improve the docking step, which is often limited by the lack of the receptor flexibility. The procedure consists in multiple docking rounds against an array of receptor structures drawn from an approximation of the Boltzmann distribution typically obtained with molecular dynamics [23]. We used the conformations resulting from Hamiltonian Monte Carlo (HMC) and molecular dynamics simulation and conducted an ensemble docking experiment.

Rescoring is most of the time based on end-point free energy estimations such as MM-PBSA or geometric methods based on input from experimental data. Before entering Stage III, a preliminary efficiency estimation may be performed. The most common way to evaluate docking efficiency is to use statistical Receiver Operator Curve (ROC) analysis after running it on a library consisting of known compounds, namely true positives (TP), and known true negatives (TN), namely decoys. As measures of accuracy, the true positive rate (TPR or sensitivity) represents the fraction of the identified positives that are true positives from all positives, while the false positive rate (fall-out or FPR or inverted specificity) is the fraction of the negatives that are FP from the total number of negatives. ROC then is the plot of TPR versus a FPR both as a function of a running threshold [24]. This kind of analysis is particularly important where the decision is probabilistic and depends on the picked threshold, method development being such a case.

APJR virtual screening studies are scarce and only focus on few compounds [15,25,26,27,28,29]. On the other hand, numerous compounds were tested using binding or activity assays such as McAnally et al. 2017 which conducted a HTS assay to screen a library of approximately 500 modified apelin peptides [30] or Chen et al., 2018, which tested 788 small compounds and ranked them according to their EC50 in a GTPγS binding assay [31].

**Figure 1:**
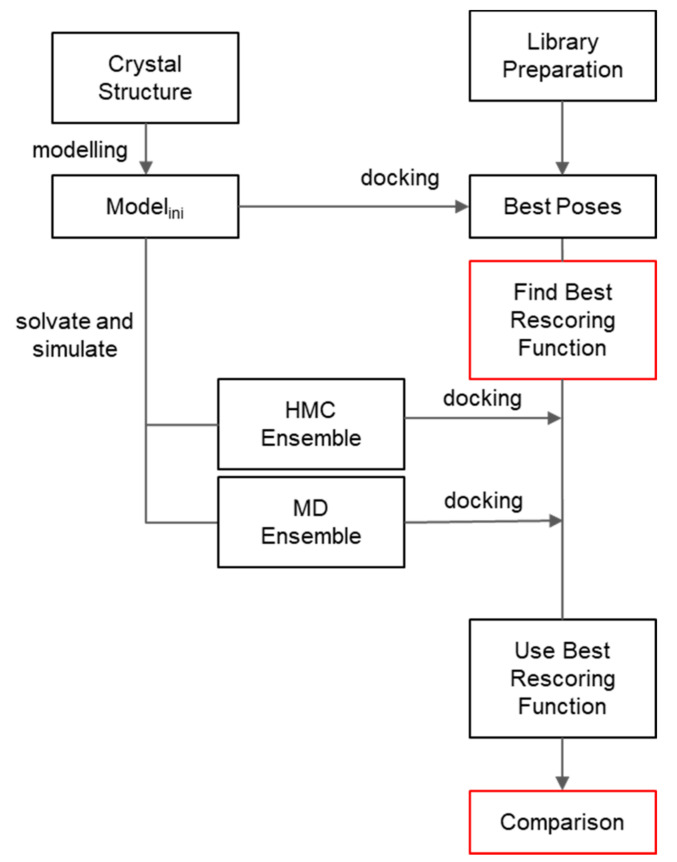
Workflow employed in current work. Red boxes indicate the stages improved herein.

Here we aim at improving any future virtual screening endeavours for APJR, using the existing HTS empirical data deposited in BindingDB [32]. To this end, we aim to touch on two checkpoints from a typical virtual screening workflow—Figure 1: Stage II primary rescoring and the ensemble generation marked with red boxes (Figure 1). We formulate a Stage II geometric score based on a physico-chemical analysis of the receptor structure performed prior to the actual workflow and on binding pose analysis after the docking stage. The performance increase is estimated by ROC statistics against empirical data generated by Chen et al., 2018 [31].

In the final stage we checked if a limited ensemble docking brings any improvement. As a side, pose analysis in this work describes binding from a molecular structure point of view which can be useful for further drug development for APJR and easy to extrapolate to other GPCRs.

## 2. Results

### 2.1. Receptor Structural Analysis

**Figure 2:**
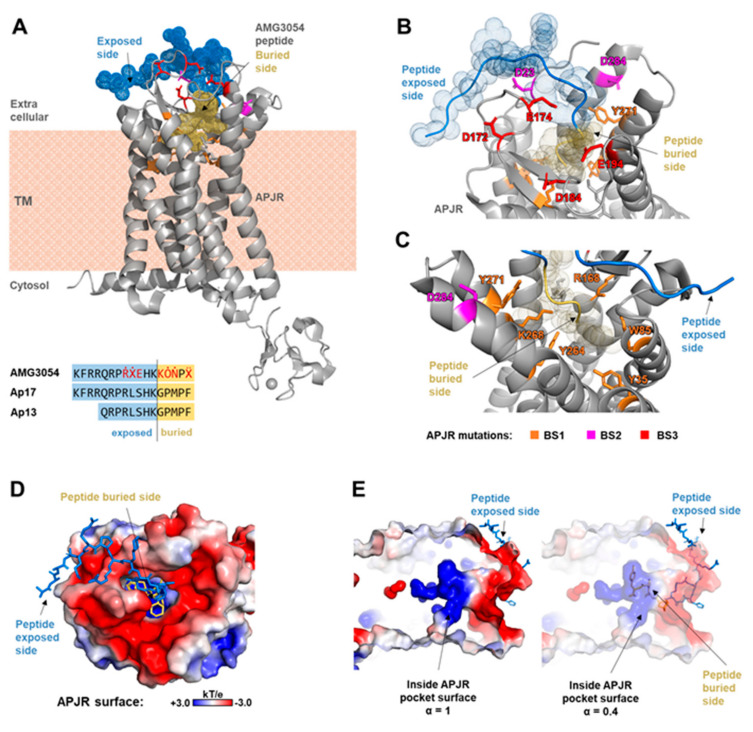
The X-ray structure of the engineered APJ receptor in complex with the mimetic peptide AMG3054 (PDB: 5VBL). (**A**) Overall view of the receptor-peptide complex. Sequence comparison of the artificial peptide AMG3054 versus apelin -13 and -17 forms is also provided. The peptide is shown as dots and colored according to its location with respect to the receptor: the exposed N-ter region (blue: aa 1–12) the C-ter end buried inside the binding pocket (yellow: aa 13–17). Modified residues in the AMG3054 peptide are Ṙ(hArg), Ẋ(Cha), Ȯ (Olc), Ṅ (Nle), Ẍ (4-Cl-Phe) [14]. (**B**,**C**) Zoom-in view of the receptor-peptide interaction site: (**B**) the acidic exterior binding site and (**C**) the interior of the binding pocket. Mutations reported in [14] to affect or totally disrupt interaction are shown with stick representation and colored according to their location on the receptor: binding sites **BS1**, **BS2** and **BS3**. (**D**,**E**) Electrostatic potential surface of the APJ receptor (blue—positively charged; red—negatively charged): top view perspective (**D**) of the extracellular side of the receptor and (**E**) inside view of the binding pocket (left: normal transparency; right—60% transparency to depict the position of the peptide inside the binding pocket). The peptide is shown in stick representation and colored as in the other panels.

**Figure A1:**
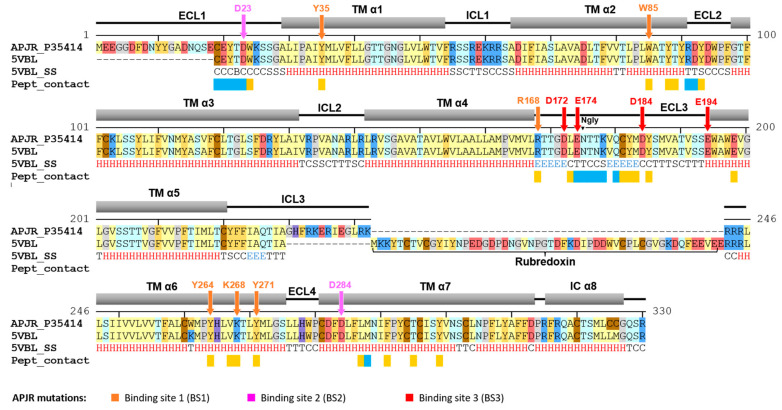
Alignment of the canonical APJR sequence (Uniprot ID: P35414) and the engineered APJR X-ray structure (PDB 5VBL). Transmembrane helices are annotated above the alignment (α1–α7), as well as the intracellular (IC) and extracellular (EC) loop (L) regions. Secondary structure assignment is displayed below the alignment (H—helix, E—beta sheet, B—beta strand, T—turn, S—bend, C—coil). Mapped are also the positions of the engineered receptor structure that are in contact (<5 Å) with the AMG3054 mimetic peptide (blue: the N-ter exposed region aa: 1–12; yellow: C-ter end buried inside the binding pocket: peptide aa 13–17). Mutations reported in [14] to affect or totally disrupt interaction are indicated above the alignment with arrows and are colored according to the binding site: orange—inside binding pocket (**BS1**), magenta—exterior pocket edge (**BS2**), red—exterior acidic groove (**BS3**). Amino acids are colored using a property color code as follows: yellow—hydrophobic aliphatic (A, V, L, I, M), orange—hydrophobic aromatic (F, W, Y), blue—positively charged (K, R), red—negatively charged (D, E), light blue—polar and neutral charge (S, T, N, Q), purple—histidine, brown—cysteine, grey—proline and glycine.

A structure of an engineered version of the human APJR has been reported in Ma et al. 2017 (PDB ID 5vbl), where in order to improve its thermodynamic stability, several point mutations were performed and a large intrinsically disordered intracellular loop ICL3 (aa 229–243) was substitute with the N-terminal domain of rubredoxin [14] (Figure A1). The engineered receptor was co-crystallized with a synthetic peptide (AMG3054) mimicking the physico-chemical properties of the endogenous ligand Apelin-13/-17 (Figure 2A–C).

The apelin receptor is a transmembrane receptor composed of 7 helical segments traversing the cell membrane. The extracellular side of the receptor consists of 4 loops (ECL) which define the entrance of the ligand towards the binding pocket (Figure 2 and Figure A1). Interestingly, the edges surrounding the ligand pocket aperture define a large negatively charged electrostatic environment, while the interior surface inside the binding pocket is highly positively charged (Figure 2D,E).

Both, the apelin-17 peptide, and its shorter form apelin-13 sequence can be roughly split into two parts: one highly positively charged (N-ter) and one hydrophobic (C-ter) (Figure 2A). The X-ray structure of the engineered APJR and AMG3054 complex [14] showed that while the C-ter half enters the receptor binding pocket (further referred to as “binding site 1 (**BS1**)”) the N-ter half of the peptide stabilizes within the exterior acidic surface of the receptor (“binding sites **BS2** and **BS3**”) (Figure 2).

Authors experimentally tested a series of mutations of the receptor residues close to the interaction site, from which we selected the ones shown to affect or totally disrupt the interaction with the peptide to be further used in our rescoring function [14] (Figure 2B,C).

### 2.2. Receptor Preparation

Considering the structural differences introduced in the engineered APJ receptor by substituting the ICL3 loop with an extraneous domain and several point mutations, we opted to build a homology model to better reflect the wild-type APJR sequence (referenced in the Uniprot database with ID: P35414), as described in methods section. We refer hereon to this model as model_ini_.

**Figure 3:**
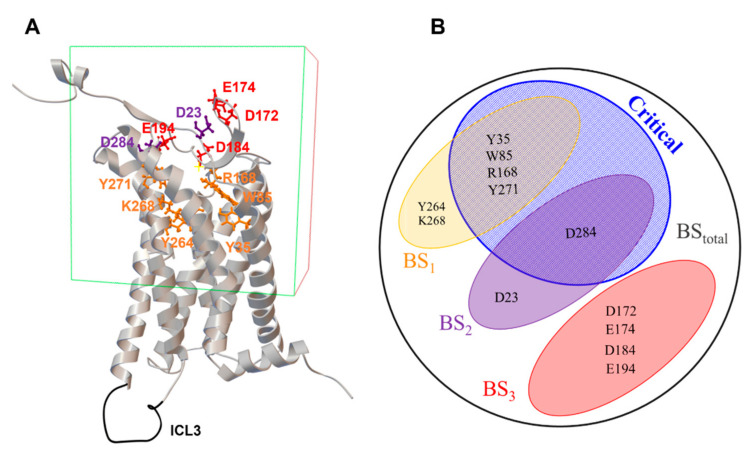
APJR grid box and binding sites definitions. (**A**) APJR Gridbox used in docking. Mutations identified by Ma et al. 2017 [14] to affect interaction are illustrated in stick representation using the color code from Figure 2. The modeled ICL3 loop is depicted in black; (**B**) Venn diagram showing the overlapping between residues in **BS1**, **BS2**, **BS3** and the critical amino acids, as identified by Ma et al. 2017 [14].

Subsequently a docking grid was generated to include all the possible contacts mentioned in [14]: T22, W24, W85, Y88, Y93, N175, T176, T177, K178, Y182, W195, Y264, K268, Y271, D284, F291, Y299 (Figure 3A). The grid covers the three sites apparent in the crystal structure and gives the library molecules an extensive search space. Also, the extensive search space allows the possibility of better separate decoys from true positives if the scoring function is geometric and focuses on a specific binding site. This is especially useful here where the natural ligand is large and only portions of it are essential to binding.

### 2.3. Ligand Library Preparation

The library was constructed from a set of 95 true positives (TP) collected from empirical based database BindingDB [32], augmented by a set of 3829 highly similar decoys generated with DeepCoy [33]. The 95 true positives (TP) were then protonated using a knowledge-based method that adds hydrogens to parts of the molecule using similarity-based searches in a database of experimentally characterized ionizable molecules [34]. The protonated TPs were then fed to decoy generation.

It’s worth mentioning here that care should be taken when producing decoys due to the unspecific basic quality of the binding site 1 as opposed to acidity of the other binding sites. Methods that change chemical properties may produce low quality decoys making a given classifier better artificially. That’s why in our case, the decoys were generated using a machine learning method implemented in DeepCoy that starts with a molecule and attempts to keep most of the physicochemical properties except the topology [33]. This forces any scoring functions to be highly specific. Even more so, the method was tested on AutoDock-GPU suite [35] which is also used here, therefore forcing us to develop good rescoring functions.

### 2.4. Docking Pose Analysis

**Figure 4:**
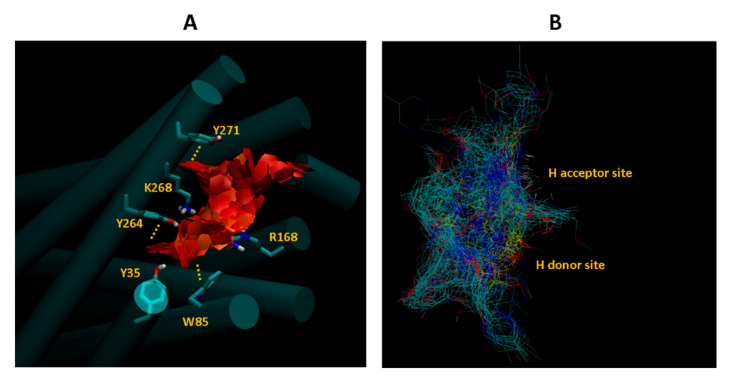
Docking pose analysis by visual inspection of the minimum energy poses clustered in the binding site. (**A**) Aromatic stacking interactions inside the binding pocket of APJR. (**B**) Hydrogen bonding interaction. Receptor is not represented.

After a docking experiment against the initial model (model_ini_) with 300 poses for each ligand, the minimum energy poses were retained for analysis. To our knowledge, the interaction of the molecules in the assay with APJR were not studied from a molecular structure point of view. This can give valuable insights into further drug development for APJR. To this end, we looked at the similarities found in the bundle of the minimum energy poses—Figure 4.

Three aromatic substructure clusters are immediately apparent from visual inspection which interact with the Y271, Y264 and W85 from the amino acids listed as important from binding in the alanine scanning assay [14]. At a closer look, tight geometric packing around the first two aromatic clusters (A1 and A2) reveal an extensive pi-stacking network formed by Y21–Y271–A1–F291–Y264–A2–Y299 (Figure 4). Even though some of the amino acids in this series are not important for apelin binding, they can be exploited for drug design purposes. In addition, from the apelin-binding amino acids list, Y35, Y264, R168, R268 act as hydrogen donors for the docked ligands while Y185, E198 act as hydrogen acceptor for most of the ligands.

### 2.5. Fast Stage II rescoring

Based on both receptor structure analysis and docking pose analysis we further analyzed the TPs in relation with the decoys. Interactions observed from pose analysis corroborates the results from the alanine scanning from Ma et al. 2017 [14] to indicate that a usual hit will bind mostly on the binding site 1 (**BS1**). The next logical step was to probe if the decoy poses maintain the same interactions. One way is to check the fitness of ligand moiety into the binding site. To estimate it numerically we devised a new score (Equation (1)) which essentially averages the minimum distances of every ligand atom to the alpha carbon of the binding site amino acids.

(1) $$s\left(\mathrm{BS}\right)=\frac{1}{n}\underset{i}{\sum }\min{\left(\left\|{\vec{r}}_{\mathrm{BS},i}-{\vec{r}}_{\mathrm{lig},j}\right\|\right)}_{j}$$

Equation (1). The new scoring function devised for preliminary Stage II rescoring or filtering based on n atoms of a defined binding site (**BS**).

**Figure 5:**
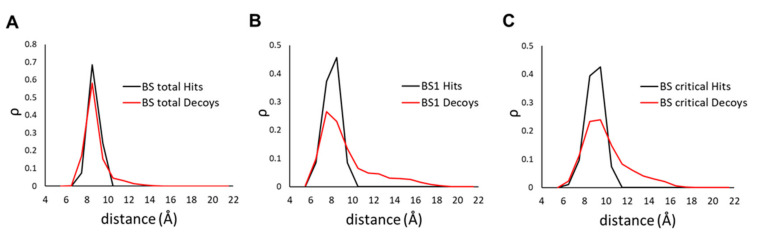
Distance distribution for the 3 binding site definitions of the TPs (black line) and decoys (red line) upon analysis of the modeled structure docking poses. (**A**) the whole binding site (**BStotal**); (**B**) binding site 1 (**BS1**); and (**C**) definition based only on amino acids that totally abolish binding (**BScritical**). Considered in the plots are the minimum distances between each ligand (any atom) and the nearest alpha carbon atom (CA) of the receptor.

The definition of the binding site for new hits, particularly the amino acids that compose it, is at best unclear and is subject for optimization. We tried 3 different binding site definitions: one based on all the three apelin binding sites: **BStot**; one based solely on site 1: **BS1**; and another one based on critical amino acids where no binding was detected upon mutation in [14]): **BScritical**. Binding site definitions are better understood as Venn diagrams (Figure 3B). We then compared the distribution of the scores for TPs and decoys as indicated in Figure 5. For simplicity we kept the same name for binding sites definitions and their scores.

**Figure 6:**
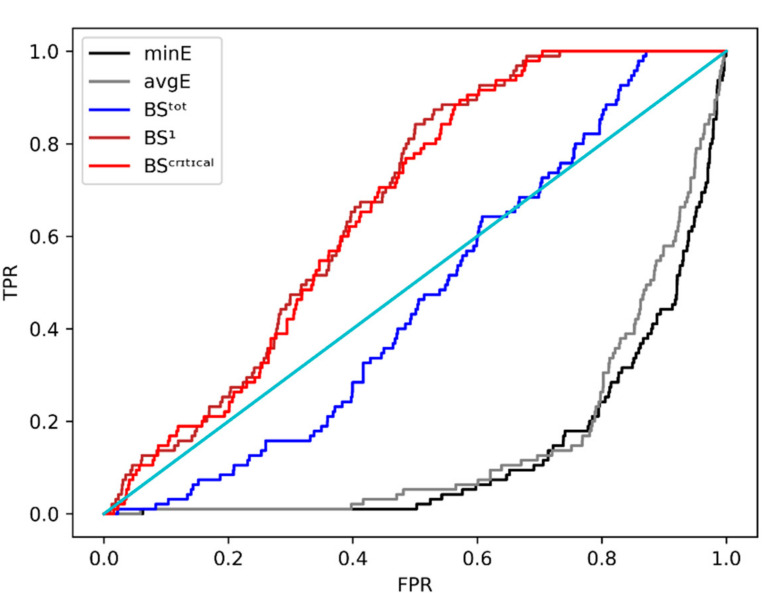
ROC curves of three different scores based on different binding site definitions (colored) and initial energy evaluation from docking against the initial model (model_lini_). minE and avgE are the minimum and the average energy of binding-based curves. **BStot** is the score based on the whole binding site, **BS1** is the score based solely on site 1; **BScritical** based on critical amino acids (no binding detected-NBD in [14]).

While the first definition does not appear to differentiate the decoys, the other two show different distributions. We further used ROC analysis which offers a more detailed characterization of the efficiency of these three scores. The ROC curves in Figure 6, more exactly, show the specificity and sensitivity of a classifier based on a particular score. While the curve itself gives a qualitative estimation of the efficiency, one numerical value that may be used to compare two ROCs is the area under their curves (AUC). Thus, the efficiency gain at rescoring was calculated as the AUC ratios of the three scores against the minimum (**minE**) binding energy from docking. We also evaluated the average energy of binding (**avgE**) which did not yield a significantly different ROC curve from the **minE**.

The ROC curves shape clearly indicate **BS1** and **BScritical** as better scores than the minimum energy of binding—**minE** given by the docking software which is not capable of distinguishing TPs from decoys. The AUC ratios in Table 1 indicate that **BS1** and **BScritical** are better than **BStot**, with a ~4-fold increase in the AUC.

### 2.6. Ensemble Docking

**Figure 7:**
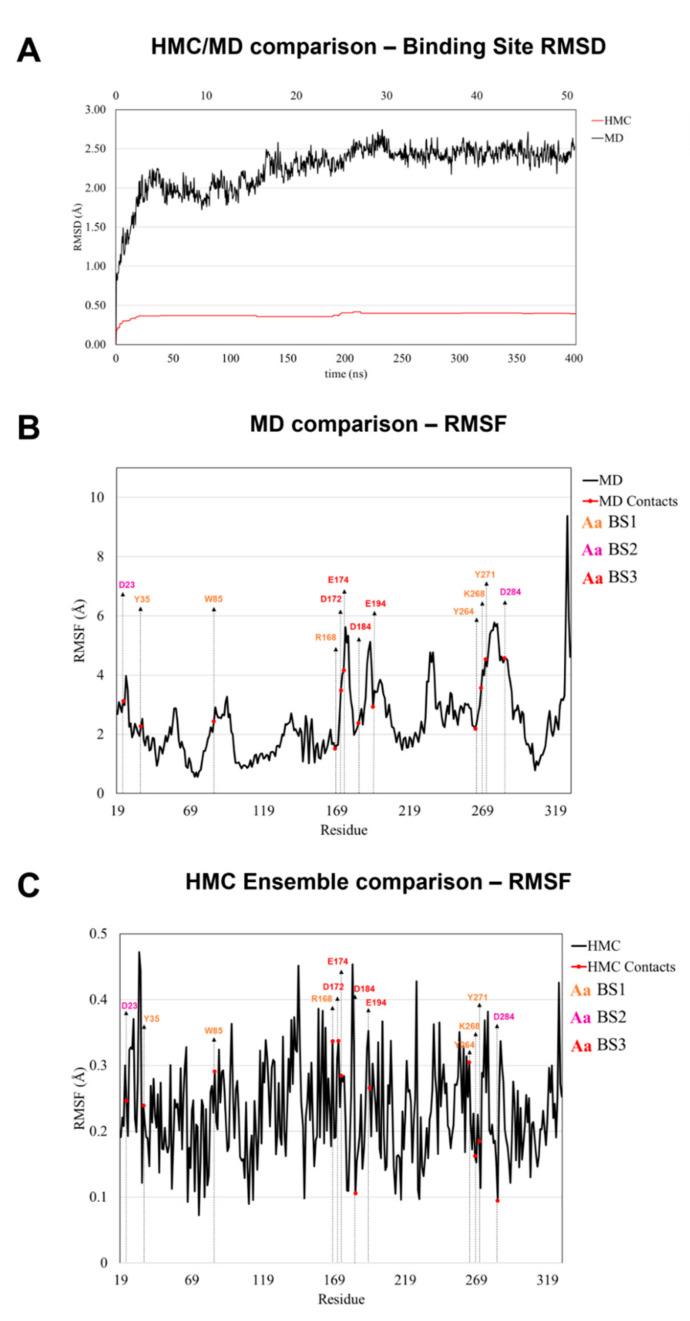
(**A**) All atom root mean square deviations (RMSD) of binding site amino acids atoms along the HMC (red)/MD (black) trajectories with respect to the initial APJR modeled structure. (**B**,**C**) Root mean square fluctuations (RMSF) using alpha carbons over the HMC and MD ensembles. Contacts with mutations reported in [14] to affect or disrupt interaction are indicated within the graph with blue circles and labeled above. The labels are colored according to the binding site, as in Figure 2.

The initial APJR receptor model was minimized after immersion in a dipalmitoylphosphatidylcholine (DPPC) membrane and solvated with 2 nm layer of water, as described under Methods. To incorporate receptor flexibility, we generated two collections of conformations around the minimum denoted as “ensembles”. This is different from regular ensemble docking procedures which aim to generate big conformational pools and use representative conformations. To generate the ensembles, the solvated system was subjected to two types of simulations: Hamiltonian Monte Carlo (HMC) and a MD (Molecular Dynamics). The HMC simulation ran for a total simulation time of ~400 ns while the MD simulation ran for ~50 ns having roughly the same number of configurations drawn. The rationale behind their lengths was to generate short simulations after reaching equilibration (Figure 7A).

The conformational diversity given by the HMC simulation appears to be low as shown by the low RMSD profile in Figure 7A, however, this might be enough to change the configuration of the binding site and affect the poses. Therefore, we tested the performance enhancement of the two ensemble docking procedures by ROC analysis by ROC analysis using **BS1** score.

**Figure 8:**
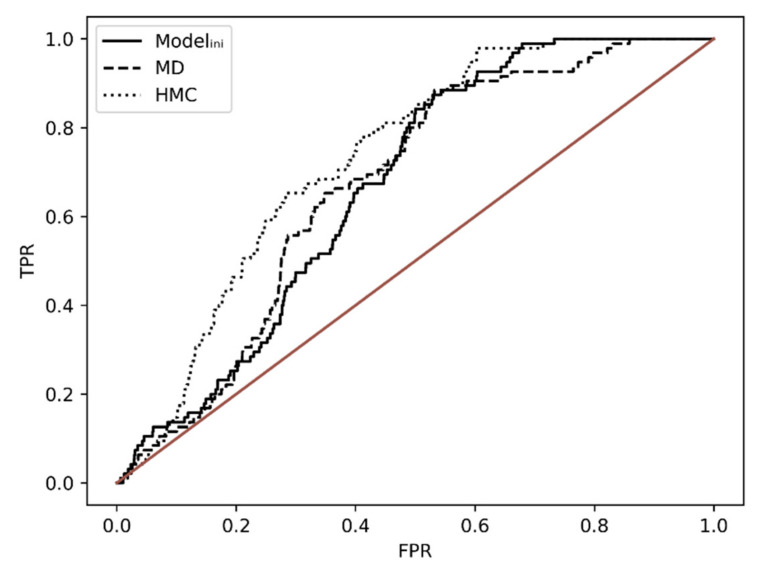
Ensemble dockings (MD and HMC) vs. initial docking (Model_ini_) ROC curves based on **BS1** score.

ROC curve shapes (Figure 8) and AUCs ratios (Table 2) indicate that both ensemble dockings, although run for a limited amount of time, performed better than the docking against a single minimized structure—Figure 8 and Table 2—and should be the preferred choice.

## 3. Discussion

We managed to find a simple and straightforward score that can be used on APJR to filter the candidates for the Stage II rescoring. The score improved the docking quality by a factor of 4 which may significantly reduce the computer effort necessary to more refined calculations such as MM/PBSA.

Short ensemble, focused mainly on side chains of the binding site, managed to increase the efficiency of docking calculations by ~10%. It is worth mentioning here that receptor and ligand preparation protocols are critical for the further steps of virtual screening such as Stage II rescoring or Stage II free energy estimation, exercising the principle of “garbage in garbage out”. This proves that prior to filtering with **BS1** score, the protocols described in this paper may be readily used for other screening studies and possibly extrapolated to other GPCRs.

**Figure 9:**
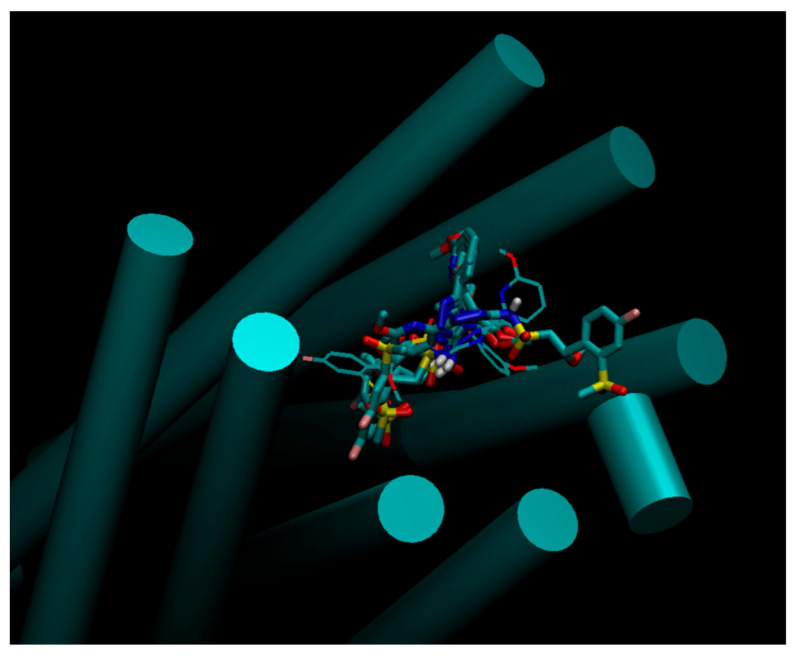
Example of ligand 930 [36] pose bundles from the HMC ensemble.

We also provided analysis of the docking poses (Figure 4 and Figure 9) pinpointing interactions that may be further exploited for developing new compound sets. Even though the protocols in this paper gave good results as indicated by the statistical analysis, large conformational changes such as “breathing” motion of membrane receptors are extremely hard to sample unless using an enhanced sampling technique such as parallel tempering. One common way to obtain such motions is to use long molecular dynamics simulations, with the note that they only approximate the Boltzmann distribution.

The downfall of any virtual screening protocol based on docking, including ensemble docking, although it explores the conformational space of the receptor, does not include the induced fit model of binding.

## 4. Materials and Methods

### 4.1. Molecular Modelling

A homology model of the human canonical APRJ (UniprotID: P35414) was generated starting from the structure of an engineered apelin receptor as a reference structure (PDB: 5vbl) [14]. The model was generated using Modeller version 9.21 [37] using the following procedure: conserved regions were modeled by coordinate transfer whereas the ICL3 loop (aa 229–243) was generated randomly and filtered for steric constraints followed by sidechain reconstruction. Further optimization included sidechain minimization with harmonic restraints using a constant of 10 kcal/nm^2^ followed by loop and overall system energy minimization. Model quality was assessed using Molprobility [38] v.4.5.1 score 0.98.

The minimized protein was inserted into a 10.7 nm by 10.6 nm dipalmitoylphosphatidylcholine (DPPC) membrane using OPM method which minimizes the water-membrane transfer free energy [39] and solvated using a 10.98 nm height TIP3P water box using CHARMM-GUI Membrane Builder [40,41,42,43,44,45,46].

The system net charge was neutralized with Na and Cl ions. The overall solvated system consisting of 94,538 atoms (protein, lipids and water molecules) was further optimized by iterative rounds of energy minimization, heating and equilibration using OpenMM v7.4.2 [47].

After the minimization stage, the system was heated from 0 to 300 K in incremental steps over a simulation time of 1 ns and followed by an equilibration stage of 3 ns at constant 300 K temperature and 1 bar pressure and a surface tension of 200 bar nanometer, using a Langevin integrator with a 2 fs step and Monte Carlo Membrane Barostat as implemented in OpenMM. Periodic boundary conditions (PBC) were applied and long range nonbonded interactions were computed using the Particle Mesh Ewald (PME) algorithm [48] with a cutoff of 1.2 nm. The simulations used Amber ff14SB [49] force field for proteins and lipid17 [50] and gaff2 for membrane [51].

### 4.2. Molecular Simulations

MD simulation was performed using OpenMM v7.4.2 [47]. Trajectory propagation assumed NpTγ ensemble using Monte Carlo Membrane Barostat algorithm implemented in OpenMM with anisotropic pressure coupling. Configurations were retrieved every 25,000 steps, using an integration step of 0.002 ps. Total simulation time was 50.8 ns. A structure was drawn every 253 frames from the simulation and retained for ensemble docking. The first frame was dropped as equilibration. The whole simulation lasted roughly 2 days on a regular desktop computer (2 × GPU GeForce GTX 1080Ti and CPU IntelI i7-8700).

HMC simulation [17,52] was performed using OpenMM v7.4.2 [47] and trajectory propagation assumed NpTγ ensemble using Monte Carlo Membrane Barostat algorithm implemented in OpenMM to obtain anisotropic pressure coupling. Samples were retrieved every 25,000 acceptance-rejection steps each of 10 MD steps using an integration step of 0.002 ps. Total simulation time was 402.3 ns. A structure was drawn every 201 frames from the simulation and retained for ensemble docking. The first frame was dropped as burn-in. The whole simulation lasted approximately 8 days on a regular desktop computer (2 × GPU GeForce GTX 1080Ti and CPU InI(R) i7-8700).

### 4.3. Library Assembly

From a total of 4788 APJR binding ligands existing in BindingDB [32], 788 were selected from Chen et al., 2018 GTPγS binding assay [31]. From this subset, 95 ligands, with an EC50 value < 1.0 nM, were further used as true positives in docking experiments and to generate a ligand set of decoys using DeepCoy [33]. The final ligand library comprises a total of 3924 compounds: 95 true positives and 3829 decoys.

The compounds were protonated using Dimorphite-DL [34] at a pH of 7.4 and pkA standard deviation of 0.1. Decoys were generated with DeepCoy [33]. The 3D structures were generated starting from the protonated molecules as SMILES strings, using Balloon 1.8.0 [53]. Atom types were assigned with Open Babel 2.3.2 [54,55]. Ligands were parameterized with Generalized Amber Force Field 2 (GAFF2) [51] using AmberTools 16 [56] and assigned AM1BCC partial charges at the same pH using Antechamber from the Amber16 package [56].

### 4.4. Docking

The APJR minimized modeled protein structure, the MD and the HMC ensembles were used for docking experiments after conversion to pdbqt format using Open Babel 2.3.2 [54,55]. The docking gridbox parameters were estimated as the maximum confining box of potential contacts defined by T22, W24, W85, Y88, Y93, N175, T176, T177, K178, Y182, W195, Y264, K268, Y271, D284, F291, Y299 residues [14]. The resulting gridbox was centered at the mass center of the set of residues aforementioned and had 115, 73 and 117 points on X, Y, Z axes, respectively, with a spacing of 0.375.

Docking was performed with the Autodock GPU v1.3 [35] which generated 300 poses per ligand for the initial structure and 100 poses per ensemble configuration using Lamarckian Genetic Algorithm (LGA) [57].

### 4.5. Analysis

Most of the data analysis, including ROC, was performed using in-house developed scripts. Graphics showing protein structural representations were computed using PyMOL [58] and VMD [59]. Electrostatic potential computations were performed using the APBS method [60] in PyMOL. Data plots were generated using Matplotlib [61] and MS Office software.

## 5. Conclusions

We presented here a rigorous and robust ensemble docking procedure for future virtual drug screening studies against APJR. The procedure implies the usage of Hamiltoniam Monte Carlo and Molecular Dynamics for short ensemble docking and a newly devised scoring function capable of differentiating non-binders from binders 4 times better than the primary docking score.

## Figures and Tables

**Table 1:** AUC for different scores (first column) and their respective ratio with the docking minimum energy.

	AUC	AUC Ratio
minE	0.142	1
avgE	0.164	1.154
BS^tot^	0.464	3.267
BS^1^	0.664	4.676
BS^critical^	0.656	4.619

**Table 2:** AUC for rescoring of the docked ligands against the minimized structure and the ensembles ones. Model_ini_ AUC is taken to be the reference for the AUC ratios.

	AUC	AUC Ratio
Model_ini_	0.664	1.000
MD	0.665	1.001
HMC	0.725	1.091

## Data Availability

Chemical structure data were retrieved from PDB and BindingDB: www.pdb.org (accessed on 15 December 2020), https://www.bindingdb.org (accessed on 29 April 2021) respectively.

## Sample Availability

Not available.
